# TLR1-10, NF-κB and p53 expression is increased in oral lichenoid disease

**DOI:** 10.1371/journal.pone.0181361

**Published:** 2017-07-17

**Authors:** Peter Rusanen, Emilia Marttila, Johanna Uittamo, Jaana Hagström, Tuula Salo, Riina Rautemaa-Richardson

**Affiliations:** 1 Department of Bacteriology and Immunology, University of Helsinki and Helsinki University Hospital, Helsinki, Finland; 2 Department of Oral and Maxillofacial Diseases, University of Helsinki and Helsinki University Hospital, Helsinki, Finland; 3 Research Unit on Acetaldehyde and Cancer, University of Helsinki, Helsinki, Finland; 4 Department of Pathology, University of Helsinki and Helsinki University Hospital, Helsinki, Finland; 5 Cancer and Translational Medicine Research Unit, University of Oulu, and Medical Research Centre Oulu University Hospital, Oulu, Finland; 6 Division of Infection, Immunity and Respiratory Medicine, Faculty of Biology, Medicine and Health, University of Manchester; and University Hospital of South Manchester, Manchester, United Kingdom; University of Pittsburgh, UNITED STATES

## Abstract

Toll-like receptors (TLRs) and nuclear factor-κB (NF-κB) in keratinocytes play an important role in dermatological autoimmune diseases. Tumour suppressor protein p53 regulates TLR expression. The aim of this study was to compare the expression of TLR1-TLR10, p53 and NF-κB in patients with oral lichenoid disease (OLD) with healthy mucosa. Oral mucosal biopsies from 24 patients with OLD and 26 healthy controls (HC) were analysed for the expression of TLR1-TLR10, NF-κB and p53 by immunohistochemistry. The expression of all TLRs was increased in OLD epithelia compared to HC samples and the difference was significant in TLR1, TLR3, TLR4, TLR5, TLR6 and TLR7. In the basement membrane zone, the immunoreactivity of TLR5 was significantly more intense in OLD compared to HC. In the intermediate layer, the immunoreactivity of NF-κB was significantly stronger in OLD, whereas the staining for p53 was more intense in all layers of OLD compared to HC samples. In OLD, a positive correlation between TLR2 and NF-κB in the basal layer and between TLR5, p53 and NF-κB in the intermediate layers was discovered. The expression of TLRs, p53 and NF-κB is increased in OLD, which may play a role in the pathogenesis of this chronic immune-mediated mucosal disease.

## Introduction

Oral lichenoid disease (OLD) encompasses oral lichen planus (OLP) and oral lichenoid lesions (OLL) [1, 2]. OLL typically resembles a type IV hypersensitivity reaction [3] whereas OLP is an immune related disease. Despite these distinct ethiopathological features, OLP and OLL are often based on clinical or histopathological evaluation difficult to set apart. The diagnosis is therefore based on both clinical and histological findings [4]. Both OLP and OLL are classified as potentially malignant disorders [5].

Human toll-like receptors (TLRs) are a family of ten transmembranous pattern recognition receptors and a part of the innate immunity. They can be activated by microbial products and endogenous particles. They maintain tissue homeostasis by regulating the inflammatory and tissue repair responses to injury [6]. TLRs are expressed by immune system cells, such as macrophages and dendritic cells, but are also present in non-immune cells, such as keratinocytes of the skin and oral mucosa [7]. Soluble forms of TLRs have been described especially in saliva [8–10].

TLR receptor engagement activates several transcription factors, such as nuclear factor-κB (NF-κB). NF-κB controls the expression of genes encoding cytokines, chemokines and enzymes that regulate innate and adaptive immune responses [11, 12]. Some of these polypeptides activate receptors that further propagate and amplify the inflammatory response, including broader innate immune responses. Some of these polypeptides can activate also NF-κB and this type of positive regulatory loop may exacerbate and perpetuate local inflammatory reactions [13]. The activation of NF-κB and cytokines has been demonstrated to play an important role in OLP [13].

The tumor suppressor protein p53 is a transcription factor that controls cell cycle, activation of apoptosis and preservation of genetic stability [14]. p53 also regulates TLR expression and modulates the response of TLRs to their ligands [15, 16]. In response to various cellular stresses, such as DNA damage or the presence of oncogenes, virus infection and hypoxia, p53 translocates into the nucleus. In nucleus, it binds to specific DNA sequences wherein it regulates transcription of genes involved in several functions such as DNA repair and apoptosis [17, 18].

The aim of this study was to compare the expression levels and tissue localisation of TLRs 1–10, p53 and NF-κB in mucosal biopsies from patients with OLD and healthy controls. The hypothesis of this study is that the expression and the localisation of TLR, NF-kB and p53 in OLD differ from HC.

## Materials and methods

### Study design

A total of 60 patients, 30 with a clinical diagnosis of oral lichenoid disease (OLD) and 30 healthy controls (HC) treated at the Department of Oral and Maxillofacial Surgery, Helsinki University Central Hospital or at the Helsinki University Dental Hospital during 2007–2011 were enrolled to this study. Patients who had received antimicrobial therapy (i.e. antibiotics, antifungals, or antiviral agents) within the past seven days and those diagnosed with HIV or hepatitis virus infection were excluded. All study patients were generally well without any systemic diseases or immune suppression predisposing them to infection. All participating subjects signed an informed consent before inclusion. The study was approved by the Ethics Committees of the Helsinki University Central Hospital and the Helsinki Municipal Health Centre (Ethical approval number 126/E6/07 25.4.2007). The Study was performed in accordance with the Declaration of Helsinki.

### Histopathological samples

Full thickness biopsies including epithelial and stromal tissue were collected as part of routine histopathological diagnostics from OLD patients from the site of active disease process according to normal clinical procedures. The samples were fixed in 10% buffered formalin and embedded in paraffin. The diagnoses of OLP or OLL were based on the clinical and histopathological criteria provided by the World Health Organization [19] and clarified by van der Meij [20]. Of the 30 patients enrolled into the study with the clinical diagnosis of oral lichenoid disease (OLD) 24 were histopathologically confirmed as oral lichen planus (n = 10) or lichenoid reaction or lichenoid lesion (n = 14). The remaining six samples were diagnosed as hyperkeratosis (n = 4), epithelial hyperplasia (n = 1) and morsicatio (n = 1) and were excluded from the analyses. The biopsies from healthy control patients were collected from the non-inflamed, healthy buccal mucosa at the incision site immediately after surgical extraction of a retained wisdom tooth.

### Immunohistochemical staining

Tissue sections, 4μm in thickness, were prepared from the paraffin embedded samples and applied to glass slides. The deparaffination was done in xylene, followed by rehydration in graded ethanol. To expose the antigenic determinants after formalin fixation and paraffin embedding the sections were incubated in pepsin for 30min at room temperature. Endogenous peroxidase activity was quenched in the sections by incubating in hydrogen peroxidase in methanol. The TLRs were visualized using avidin-biotin-peroxidase complex method (catalogue nos., PK-4001 and PK-4005; Vectastain ABC kit; Vector Laboratories, Peterborough, England).

The optimal primary antibody concentrations for immunohistochemistry was selected based on pilot experiments. The final IgG concentrations of the polyclonal anti-human antibodies used in this study were as follows: 4μg/ml rabbit TLR1, 4μg/ml goat TLR2, 4μg/ml rabbit TLR3, 4μg/ml rabbit TLR4, 4μg/ml rabbit TLR5, 4μg/ml rabbit TLR6, 5μg/ml rabbit TLR7, 4μg/ml rabbit TLR8, 5μg/ml rabbit TLR9, 5μg/ml rabbit TLR10 (catalogue nos., sc-30000, sc-8689, sc-10740, sc-10741, sc-10742, sc-30001, sc-30004, sc-25467, sc-25468, sc-30198; Santa Cruz Biotechnology, Santa Cruz, California, USA). Control incubations were performed by replacing primary antibodies with protocol buffer. Sections from each sample were also stained with periodic acid-Schiff (PAS) to determine the presence or absence of secondary candidiasis.

For the immunohistochemical staining with NF-κB, the tissue sections were buffered in citrate, pH 6 and heated 10 minutes in microwave oven and incubated for one hour in room temperature with an optimally diluted NF-κB antibody. For the immunohistochemical staining of p53, the tissue sections were buffered in Tris-EDTA, pH 9 and heated 15 minutes in microwave oven and incubated for 30 minutes RT with an optimally diluted p53 antibody. After the primary antibody incubation, the tissue sections were incubated separately with Dako REAL™ EnVision™ kit using Dako automated immunostaining instruments. The reactions were visualized by Dako REAL™ DAB+ Chromogen also included in the kit (catalogue number K5007, Dako Glostrup Denmark). The concentrations of NF-κB and p53 IgG antibodies used in this study were as follows: 1:150 polyclonal rabbit anti-human NF-κB (catalogue number, sc-114; Santa Cruz Biotechnology, Santa Cruz, California, USA) and 1:600 monoclonal mouse anti-human p53 (catalogue number, M7001; Dako Glostrup Denmark). Control incubations were performed by replacing primary antibodies with protocol buffer.

Gingival tissue samples from patients with chronic periodontitis obtained during periodontal flap operations were used as positive controls for all immunohistological staining [21, 22].

### Microscopical analyses

The expression for TLR1-TLR10, p53 and NF-κB was analysed using a light microscope (Nikon Eclipse 80i). Results were scored semi-quantitatively and photographed using the attached camera (Nikon DS-Fi1). All samples and stainings were analysed and scored by four authors (PR, JH, EM and TS) blinded for each other’s scoring and clinical data. Discrepancies were settled within the team. The staining quantity of the basement membrane zone and of the cells in the basal, intermediate and superficial layers of the epithelium were scored in a four-point scale as 0 = no staining, 1 = staining of approximately 1–33% of cells or of the basement membrane zone, 2 = staining of 34–66% and 3 = staining of 67–100%.

### Statistical analysis

Data was analysed by GraphPad Prism version 5.00 (GraphPad Inc. San Diego, California, USA). The two-tailed Mann Whitney test and Spearman’s rho (r_S_) were used for the analyses of correlations. The Wilcoxon signed-ranks test was used to compare the differences between the different layers of samples. P-values of less than 0.05 were considered statistically significant.

## Results

### Subjects

The mean age of the included OLD patients was 53.1 years, (range 23–74). Of the patients in the OLD group, 15 were female with the mean age of 57.1 years (range 29–71), and nine were male with the mean age 46.3 years (range 23–73). The anatomical sites of OLD biopsies were the cheek (n = 17) and the tongue (n = 7). Of the 30 generally healthy patients enrolled into the study, 26 were finally included in the study as four had provided inadequate amount of sample for histopathological analyses. Of the healthy control (HC) patients included in the analyses, 17 were female with the mean age of 32.2 years (range 19–56 years) and nine were male with the mean age of 26.1 years (range 18–36 years). The mean age of all the HC patients was 30.8 years (range 18–56).

### The expression of TLR1-TLR10

**Fig 1:**
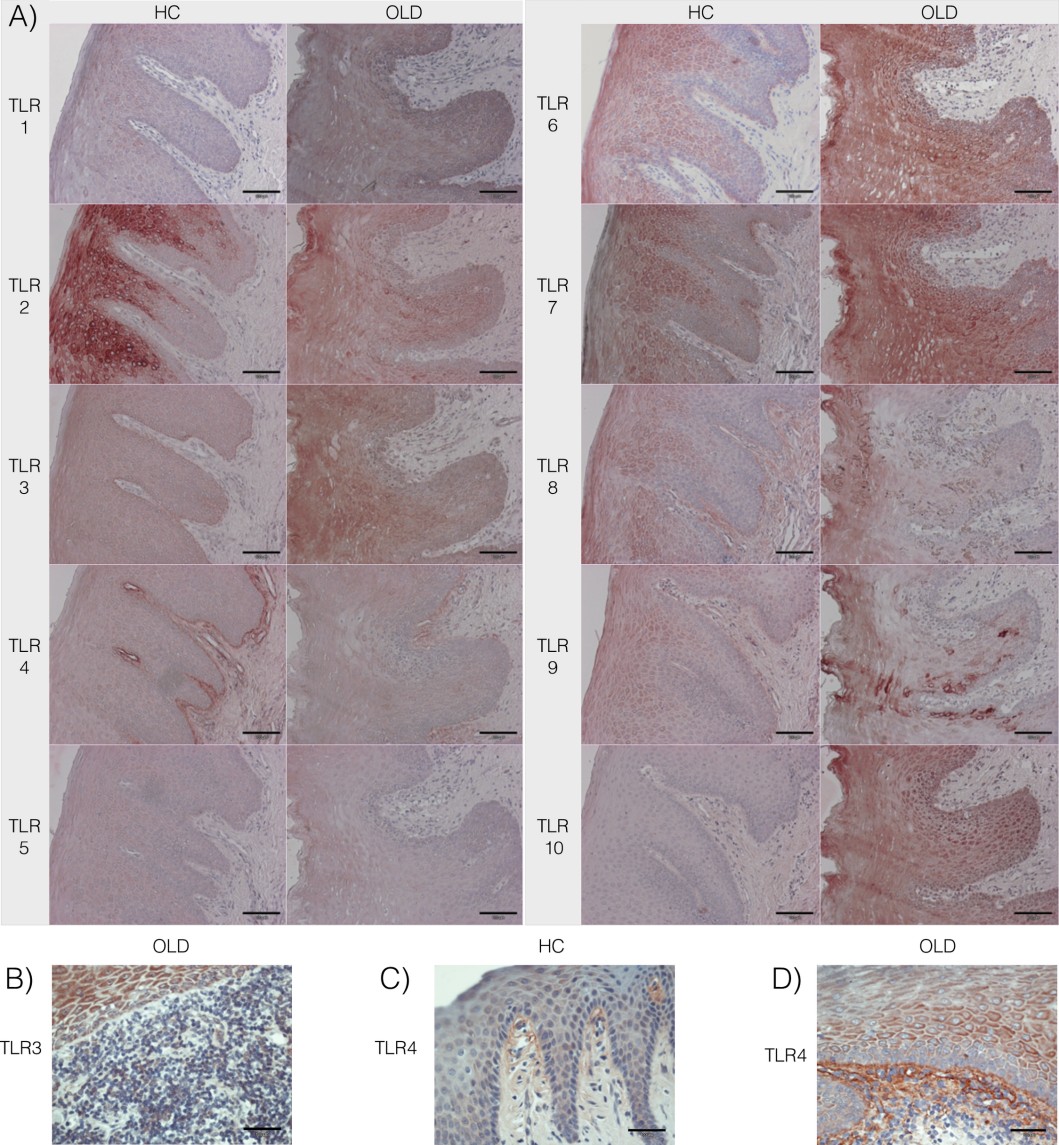
The staining for TLR1-10 in healthy control and oral lichenoid disease samples. (A) Staining of all TLRs was detected in all samples (Healthy controls, HC n = 26; Oral lichenoid disease, OLD n = 24; x20). (B) Staining for TLR3 was not detected in the basement membrane (BM) zone of OLD samples but was seen in the superficial parts of the epithelium (x40). In both, (C) HC and (D) OLD samples, staining intensity of TLR4 was significantly stronger in the BM zone compared to the other layers (x40).

All other TLRs, except TLR2, were expressed throughout oral epithelia in both OLD and HC samples (Fig 1A). TLR2 was not detected in the basal layer or the basement membrane (BM) zone of the HC samples, and was seen in only one of the OLD samples in the BM zone. Likewise, staining for TLR3 was seen throughout the intermediate and superficial layers of the OLD and HC epithelia but in only one of the OLD samples in the BM zone (Fig 1B). In contrast, the staining intensity of TLR4 was significantly stronger in the BM zone compared to the other layers in HC and OLD samples (Fig 1C and 1D). However, the expression of most of the TLRs had a trend of a gradual increase from the basal layer towards the superficial layers. Expression was strongest in the superficial layer for all TLRs, except for TLR3 and TLR4 in the HC samples, and TLR4 and TLR9 in the OLD samples.

**Fig 2:**
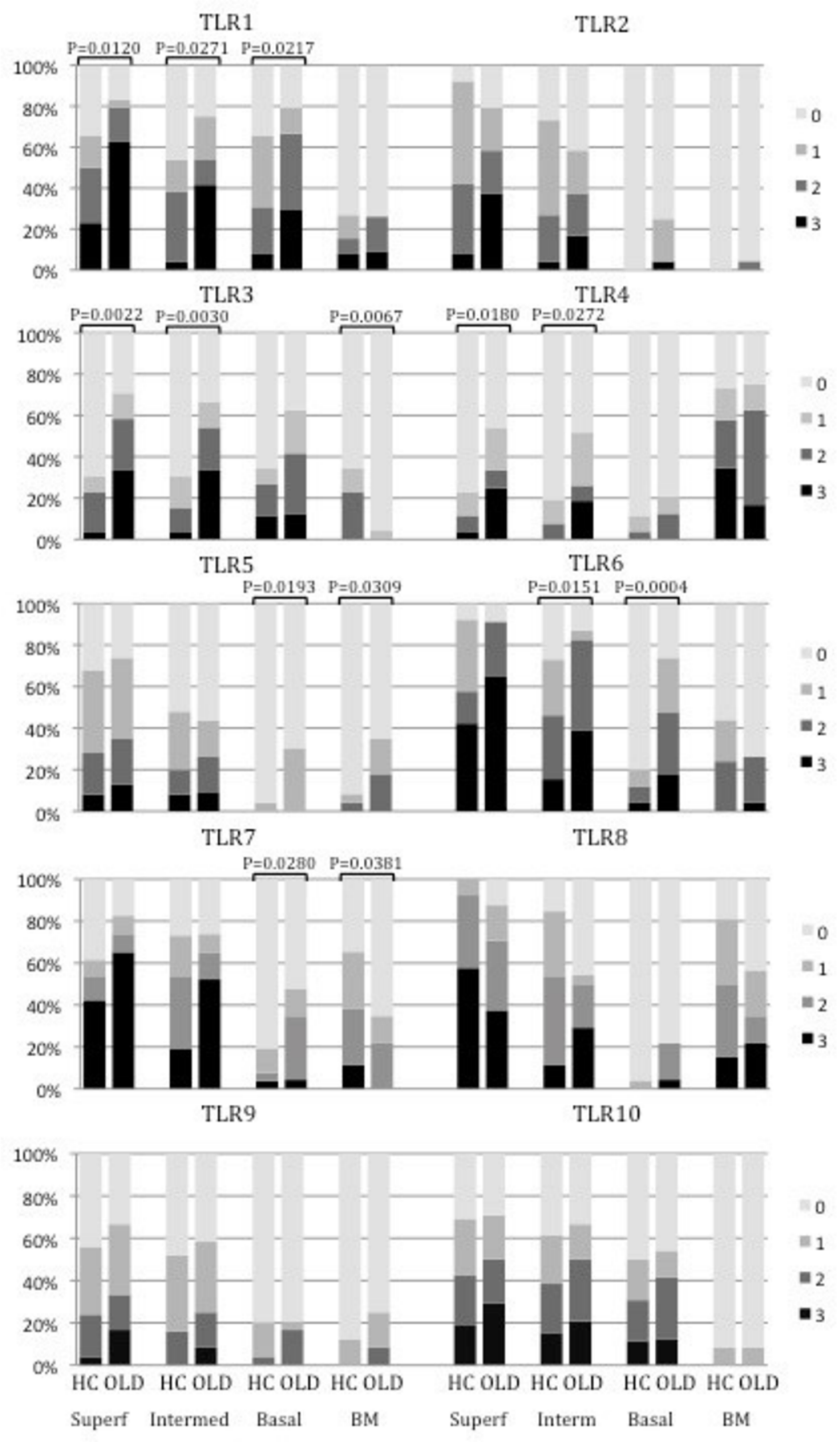
The staining percentages of TLRs in epithelial layers and basement membrane (BM) zone. In general, the expression of several TLRs was markedly upregulated in the oral lichenoid disease (OLD) samples compared to the healthy control (HC) samples (HC n = 26; OLD n = 24). 0 = no staining, 1 = staining of approximately 1–33% of cells, 2 = staining of 34–66% of cells and 3 = staining of 67–100% of cells. Bars represent means ± SEM. ***P < 0.*0001*, **P < 0.*001*, *P < 0.*05*, Mann Whitney.

In general, the expression of several TLRs was markedly upregulated in the OLD samples compared to the HC samples (Fig 2). In the superficial epithelium, the staining intensity for TLR1, TLR3 and TLR4 was significantly higher in OLD samples when compared to the HC ones (*P =* 0.01, *P* = 0.002, *P* = 0.02, respectively). The staining intensity for TLR1, TLR3, TLR4 and TLR6 was also significantly higher in the intermediate layer of OLD samples (*P* = 0.03, *P* = 0.003, *P* = 0.03, *P* = 0.02, respectively). In the basal layer, the expression of TLR1, TLR5, TLR6, and TLR7 was increased in the OLD group when compared to HC (*P* = 0.02, *P* = 0.02, *P* = 0.0004 and *P* = 0.03, respectively). In the BM zone, the expression of TLR5 was upregulated in OLD when compared to HC samples (*P* = 0.03). In contrary, the expression of TLR3 and TLR7 in the BM zone was stronger in the HC samples than in the OLD samples (*P =* 0.007 and *P* = 0.04, respectively). All inflamed gingiva control samples showed a positive staining to all TLRs. (Fig 2). The expression of TLRs. did not correlate with the age of the patients.

### The expression of p53

**Fig 3:**
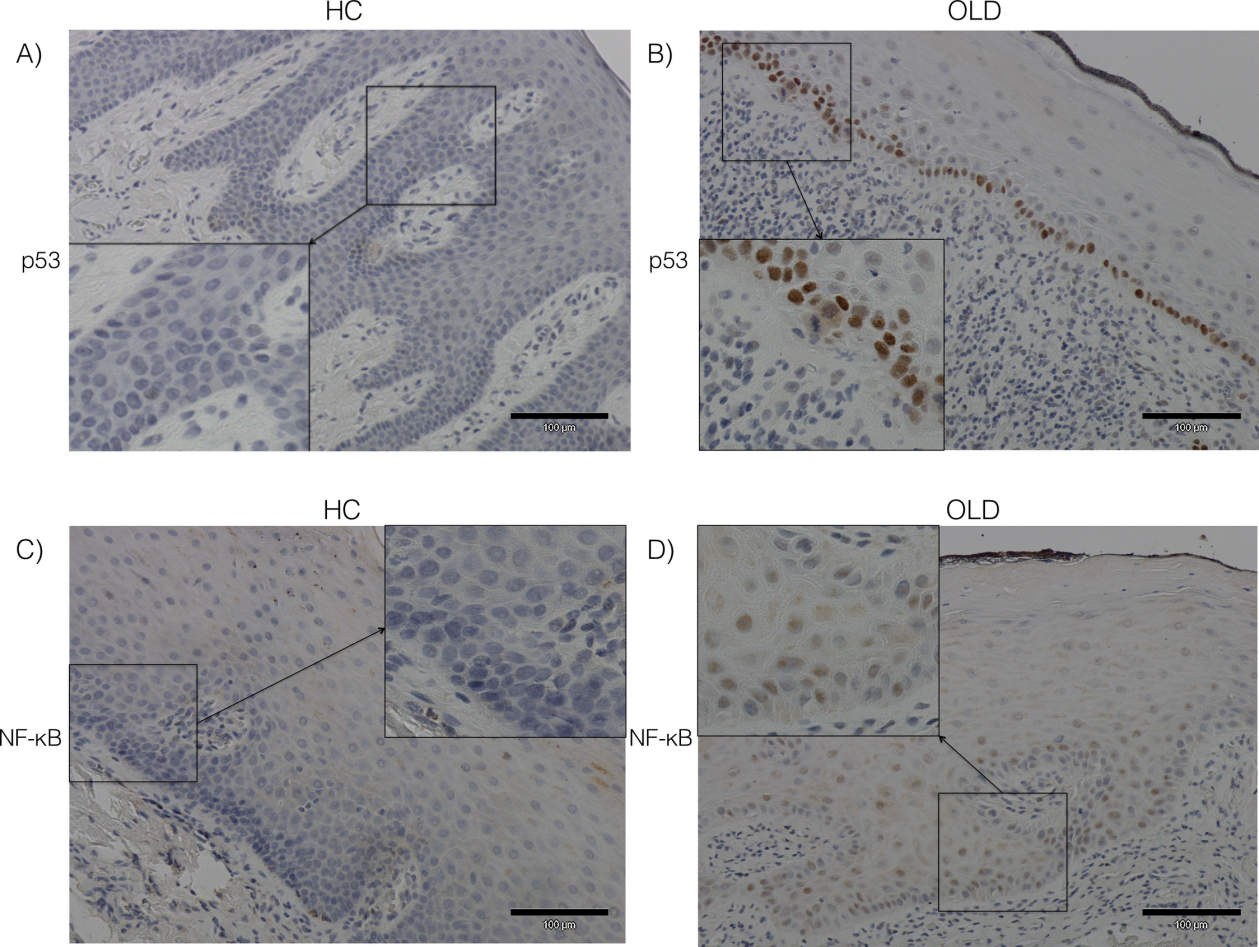
The staining of p53 and NF-κB in healthy control and oral lichenoid disease samples. (A, B) The staining for p53 decreased from the basal epithelium towards the superficial layers in both healthy control (HC) and oral lichenoid disease (OLD) samples. The staining for p53 was more intense in the OLD samples compared to the HC samples. (C, D) The staining for NF-κB increased from the basal epithelium towards the superficial layers in both HC and OLD samples. The staining for NF-κB was more intense in the OLD samples compared to the HC samples (HC n = 26; OLD n = 24; x20, inserts x40).

**Fig 4:**
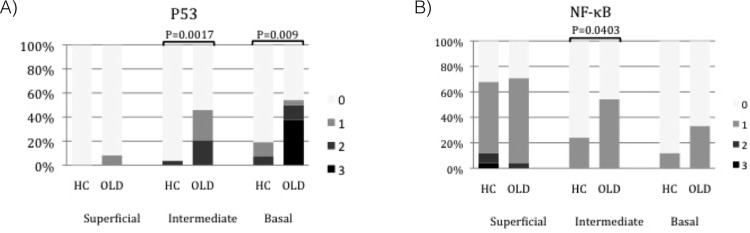
The staining percentages of p53 and NF-κB in healthy control and oral lichenoid disease samples. The staining of (A) p53 and (B) NF-κB in the healthy control (HC) samples compared to the oral lichenoid disease (OLD) samples in three different epithelial layers (HC n = 26; OLD n = 24): superficial, intermediate and basal layers. 0 = no staining, 1 = staining of approximately 1–33% of cells, 2 = staining of 34–66% of cells and 3 = staining of 67–100% of cells. Bars represent means ± SEM. ***P < 0.*0005*, **P < 0.*005*, *P < 0.*05*. Mann Whitney.

The expression of p53 decreased from the basal epithelium towards the superficial layers in both HC and OLD samples (Fig 3A and 3B). Staining for p53 could not be detected in the superficial epithelial layers of the HC samples, whereas two of the OLD cases showed weak staining (1–33% of cells) in this layer. In the HC group, staining for p53 could be detected only in one sample in the intermediate layer and in five samples in the basal layer. The immunopositivity of p53 was statistically stronger in basal layer compared to the intermediate layer in the HC group (*P* = 0.02). In OLD samples, the staining intensity was significantly lower in the superficial layer compared to the intermediate layer (*P* = 0.002) and in the superficial and intermediate layers compared to the basal layer (*P* = 0.001, *P* = 0.003, respectively). In general, the staining for p53 was more intense in the OLD samples compared to the HC samples in all layers. The most significant difference was seen between the basal and the intermediate layers (*P* = 0.002 and *P* = 0.009, respectively; Fig 4A).

### The expression of NF-κB

The expression of NF-κB increased from the basal layer towards the superficial epithelial layers in both the HC and OLD samples (Fig 3C and 3D). Expression of NF-κB could be detected in all epithelial layers in both HC and OLD samples. In the HC samples, the expression of NF-κB was significantly stronger in the superficial layer compared to the intermediate and the basal layers (*P* = 0.001, *P* = 0.0005, respectively). In the OLD samples, the expression of the NF-κB in the superficial and in the intermediate layers was significantly stronger compared to the basal layer (*P* = 0.002, *P* = 0.03, respectively). In general, the staining for NF-κB was more intense in the OLD samples compared to the HC samples. The staining intensity of NF-κB was significantly stronger in the OLD samples compared to the HC samples in the intermediate layer (*P =* 0.04; Fig 4B). However, in the superficial layer the staining of NF-κB was non-significantly stronger in the HC samples.

### The associations between the expressions of TLR1-10, p53 and NF-κB

In the basal layer of the OLD samples, the staining intensity of p53 correlated positively with the staining intensity of TLR1 (*P =* 0.02) and NF-κB (*P =* 0.02). Staining intensity of NF-κB correlated positively with the staining intensity of TLR2 (*P =* 0.05). In the intermediate layer of the OLD samples, the staining intensity of p53 correlated positively with that of TLR1 (*P* = 0.009), TLR5 (*P* = 0.03) and NF-κB (*P* = 0.03). However, no significant correlations were seen in the staining intensities of p53, TLR1-10 and NF-κB in the superficial layers of the OLD samples or in any layers of HC samples. There was no significant difference in the staining intensity for TLR1-10, p53 nor NF-kB in patients with oral lichen planus (OLP) and patients with a mucosal lichenoid reaction or lichenoid lesion in any parts of the epithelium.

## Discussion

In the present study, we found increased staining intensity for all TLRs throughout the epithelium of OLD compared to HC samples. In cases of TLR1, TLR3, TLR4, TLR5, TLR6 and TLR7 in the epithelium, and for TLR5 in the basement membrane zone, the differences were significant. The immunoreactivity of NF-κB was significantly stronger only in the OLD intermediate layers, whereas in all OLD epithelial layers, p53 staining was stronger compared to HC samples. Additionally, we found a positive correlation between TLR2 and NF-κB staining in the basal layer, and between TLR5, p53 and NF-κB in the intermediate layer of OLD. Our results suggest that TLRs, p53 and NF-κB may play role in the pathogenesis of OLD.

Significant increase of TLR2, TLR4 and TLR9 has also previously been found in OLP compared to HC [23–25]. However, unlike Ohno et al. [23], Janardhanam et al. [25] found only a minor increase of TLR2 in OLP. The discrepancies between these studies may be attributed to the differences in the antibodies and the methodology used. In the study of Siponen et al [24] a strong expression of TLR4 in the basal layer of the OLP samples was reported whereas in our study the staining of TLR4 in OLD samples was weak in the basal layer but strong in the BM zone. This difference may be due to different scoring: BM zone was recorded separately only in our samples.

The role of TLRs in the BM zone deserves more attention, since we found differences in the staining intensities of several TLRs in this area. Significant differences for TLR3, TLR5 and TLR7 between the BM zone and the basal layer were also seen between the HC and OLD groups. All the other TLRs, except TLR3 and TLR7, showed stronger staining intensity in the BM zone in our OLD samples. The weak staining of TLR3 is in accordance to the study of Sinon et al. [26] who reported a down-regulation of TLR3 in OLP. Interestingly, soluble forms of several TLRs have been detected in body fluids, such as saliva, breast milk and plasma [8–10, 27, 28]. It is therefore likely that the staining of several TLRs in the BM zone is due to the presence of soluble TLRs fragments produced by the basal epithelial cells.

Ge et al. [29] showed significantly higher expression of NF-κB in OLP than HC samples. In our cases, the expression of NF-κB in the superficial part of the oral epithelium was generally stronger in the HC than in the OLD samples, but in contrast to the superficial part, the intermediate and basal cell layers of the OLD samples showed a markedly higher staining intensity of NF-κB compared to HC. Consistent to previous studies, p53 stained significantly more strongly in OLD samples than in HC in the intermediate and basal cell layers [30, 31]. Wild-type p53 acts as a tumour suppressor and inactive p53 leads to cellular damage [18]. Many p53 mutations with altered functions can also modulate the expression of TLRs [17, 32]. The half-life of the wild-type p53 protein is short and can be difficult to detect by immunohistochemistry. However, the mutated, inactive p53 protein is more stable but it cannot be distinguished from the wild-type by immunohistochemistry. Therefore, it is not possible to say whether the strong staining for p53 seen in our study is due to overexpression of the active, wild-type p53 or to the presence of the more stable, mutated inactive p53 protein. Also, the overexpression of p53 in OLD samples is thus not necessarily associated with malignant transformation in the absence of knowledge of its biological behaviour [33].

In contrast to OLD samples, we did not find any correlations between the TLRs and other proteins analysed in HC samples. Interestingly, although there was no difference in the staining intensity for TLR5 between OLD and HC groups, there was a positive correlation between TLR5, p53 and NF-κB in the intermediate layer of OLD samples. This may reflect a change in the function of TLR5 e.g. due to a polymorphism that would induce the expression of p53. According to the study of Rutkowski and Conejo-Garcia [33] TLR5 mediated recognition of commensal microbiota can modulate the systemic tumour-promoting inflammation and malignant progression due to the polymorphism of TLR5. Kauppila et al. [34] described the role of TLR5 in the pathophysiology of oral tongue squamous cell carcinoma (OTSCC) and concluded that it could be a useful marker for predicting recurrence or survival of OTSCC patients, whereas in the study by Mäkinen et al. [35] TLR5 was not a predictive marker for OTSCC, unlike TLR2, TLR4 and TLR9. It would be of interest to conduct a longitudinal study to investigate whether an association could be found between the upregulation of NF-κB, p53, TLR1 and TLR5 and underlying immunological changes possibly leading to the malignant transformation of OLD.

In our study, the OLD patients were significantly older than the healthy controls (53.1 vs 30.8 years, *P*<0.0001) as OLD affects mainly middle-aged or older whereas surgical removal of third molars is mainly performed in young adults. This control group was chosen, as we were unable to identify an older group of healthy patients with healthy oral mucosae undergoing surgical oral procedures at our hospital. We acknowledge this as a potential limitation of this study although, in contrast to adaptive immune responses, the innate responses are not generally significantly affected by aging [36]. In fact, in our study, the expression of TLRs did not correlate with the age of the patients. However, there is evidence for impaired TLR induced downstream signalling in aging cells [37]. In addition, individual medical histories and differences in diet and life style may impact individual results, but these factors are unlikely to explain any significant differences between the two groups. In addition, they are practically impossible to control for in a clinical cross-sectional study like ours.

The main limitation of this study is that only immunohistochemistry was used to compare the level of expression of TLR, NF-kB and p53 between the OLD and HC samples due to limited amount of tissue available. In order to determine the aetiology of OLD, the factors leading to T-cell accumulation and the changes in chemokine, chemokine receptor and adhesion molecule expression should ideally be analysed using additional techniques, such as measuring mRNA levels by qPCR. However, qPCR does not provide information of the specific site within the tissue sample resulting the signal. To identify microbiomial triggers initiating the innate inflammation, the association between TLRs and specific microbial antigens should be investigated. However, it is difficult if not impossible to control for other confounding factors such as diet, oral hygiene and life style in an *ex vivo* study model. In addition, we focused on the changes in innate immunity response in OLD keratinocytes, and changes in other cells, like mast cells, were not studied. However, using the tissue collected for routine histopathological diagnostics, we were here able to map, for the first time, all TLRs, p53 and NF-κB, and their co-localization in the epithelium and BM-zone. These immunohistological findings, together with previous studies, suggest that innate inflammatory reactivity has a predominant role in OLD. This is supported also by growing evidence based on gene expression analyses [38]. An increased expression of several TLRs in OLD suggests their important role and possible treatment target in this chronic mucocutaneous disease. The role of soluble TLR forms in the BM zone calls for further studies.
